# Neck CT angiography in acute stroke: An open window for fast detection of COVID-19 lung involvement? Applicability in telemedicine

**DOI:** 10.1371/journal.pone.0281955

**Published:** 2023-02-24

**Authors:** Jorge Uclés, Emilio Cuesta, Ricardo Rigual, Jorge Rodríguez-Pardo, Gerardo Ruiz-Ares, Pedro Navía, Andrés Fernández-Prieto, Alberto Álvarez-Muelas, María Alonso de Leciñana, Blanca Fuentes

**Affiliations:** 1 Department of Neurology and Stroke Center, Hospital La Paz Institute for Health Research-IdiPAZ (La Paz University Hospital-Universidad Autónoma de Madrid), Madrid, Spain; 2 Department of Radiology, Hospital La Paz Institute for Health Research-IdiPAZ (La Paz University Hospital-Universidad Autónoma de Madrid), Madrid, Spain; Carol Davila University of Medicine and Pharmacy, ROMANIA

## Abstract

**Background:**

Chest CT has been proposed as a screening test to rule out SARS-CoV-2 lung infection in acute stroke. Our objectives are to analyze the predictive value of neck CT angiography (CTA) source images compared with conventional chest CT, the interobserver concordance and the reliability of the diagnosis using a mobile app.

**Methods:**

A retrospective observational study that included acute stroke patients admitted to a stroke center. Two raters blinded to the clinical data evaluated and classified the pulmonary findings in chest CT and neck CTA source images according to the COVID-19 Reporting and Data System (CO-RADS). CTA findings were evaluated using a conventional workstation and the JOIN mobile app. Scores of 3–5 were grouped as appearing typical or indeterminate for COVID-19 lung involvement and 0–2 as appearing atypical or negative for pneumonia. SARS-CoV-2 infection was confirmed by polymerase chain reaction (PCR).

**Results:**

A total of 242 patients were included (42 with PCR-confirmed COVID-19). In the cohort of 43 patients with both neck CTA and chest CT, the predictive value for COVID-19 was equivalent (sensitivity, 53.8%; specificity, 92.9%). The interobserver agreement in the classification into CO-RADS 3–5 or 1–2 in CTA was good (K = 0.694; standard error, 0.107). In the cohort of 242 patients with neck CTA, the intraobserver agreement between the workstation and the JOIN app was perfect (K = 1.000; standard error 0.000).

**Conclusions:**

Neck CTA enables the accurate identification of COVID-19-associated lung abnormalities in acute stroke. CO-RADS evaluations through mobile applications have a predictive value similar to the usual platforms.

## Introduction

The need to confirm or rule out SARS-CoV-2 infection during acute stroke management in order to activate the specific pathways for infected patients might compromise the speed of workflows in stroke care protocols in the pandemic era. The COVID-19 pandemic is far from over; however, in many countries reverse transcription polymerase chain reaction (RT-PCR) testing is being eliminated from general screening in emergency settings or replaced with rapid detection methods such as point-of-care antigen tests, which are not universally available. According to a recent review from the Cochrane Library, the average sensitivity for the detection of SARS-CoV-2 infection using rapid tests in asymptomatic patients is 54.7% (95%CI 47.7‒61.6%), with wider confidence intervals depending on the brand, which may lead to misdiagnosis in some cases. In fact, none of the assays evaluated by the Cochrane Library met the World Health Organization acceptable performance standards. This translates into one in two or two in three asymptomatic COVID-19 cases missed when diagnosis is made based on point-of-care antigen tests [1]. Asymptomatic clinical forms of COVID-19 still place health workers and other patients at risk of contagion. Therefore, any clinical tool included in current management protocols that can rapidly detect signs of infection could help ensure protective measures against SARS-CoV-2 infection. Numerous initiatives have been proposed to improve the sensitivity of screening strategies as well as the speed of the diagnosis [2].

Expert consensus has recommended chest CT as an additional test in acute stroke to identify radiological abnormalities suggestive of COVID-19 [3–5]. However, a delay in door-to-puncture times associated with performing chest-CT has been reported [6].

CTA is one of the most commonly used non-invasive diagnostic tests for patients with acute ischemic stroke and suspected large vessel occlusion on arrival at the emergency room [7, 8]. Source images of this test cover the lung apex, together with the thoracic and cervical vessels [9]. Incidental COVID-19-related lung apical findings on CTA have been reported in up to 37.5% of patients with acute confirmed stroke [10].

Telemedicine and mobile apps are progressively being incorporated into stroke protocols for the rapid communication and visualization of images, with positive results and excellent acceptance by practitioners. Several studies have shown good interobserver concordance and no differences in the accuracy of diagnosis between conventional radiology workstations (PACS) and these apps, as well as an acceleration of stroke code workflows when using the apps [7–10]. These apps might also enable the rapid identification of COVID-19-related lung findings in neck CTA source images, which could contribute to the fast identification of patients with COVID-19.

Our aim was to evaluate whether the assessment of lung apices usually included in neck CTA, performed as part of the routine diagnostic workup on arrival at the emergency department in patients with acute stroke, provides reliable information on the probability of an underlying COVID-19 infection. Our secondary aim was to evaluate whether the assessment of lung apices in neck CTA using the mobile app, JOIN, is comparable to the evaluation performed in the typical workstation.

## Methods

We conducted a retrospective, single-center cohort study that included patients with acute stroke admitted to a European Stroke Organization-certified stroke center.

Inclusion criteria were as follows: aged older than 18, diagnosis of acute stroke, and undergoing a neck CTA upon arrival to the emergency room as part of the acute stroke diagnostic workup. We excluded patients transferred from other hospitals who had already undergone neck CTA at the referring hospital, those with a clinical diagnosis of COVID-19 without PCR confirmation, as well as those with no apical lungs visible in the neck CTA. Confirmed diagnoses of COVID-19 were based on detecting SARS-CoV-2 nucleic acid by polymerase chain reaction (PCR) assay from nasopharyngeal/oropharyngeal swabs.

The study was conducted in two separate stages. The first stage, which included patients from the first pandemic wave (from February 25 to April 25, 2020), evaluated the intraobserver and interobserver concordance between chest CT findings and CTA for the diagnosis of COVID-19-related lung findings using conventional workstations. The second stage, which added patients with CTA from the second pandemic wave (from July 10 to November 25, 2020), analyzed the concordance between the diagnosis of COVID-19-related lung findings in CTA source images created in the conventional workstations or using a mobile app.

Lung findings were classified using the COVID-19 Reporting and Data System (CO-RADS) [11]. Scores of 3–5 were grouped as typical or indeterminate appearance COVID-19-related findings, and scores of 1–2 were grouped as atypical appearance or negative for pneumonia findings [12]. For the first stage of the study, CTA and chest CT images were independently evaluated by a radiologist specializing in chest CT diagnosis and a medical student who received a 2-hour training session in CO-RADS evaluation from the expert radiologist. The student also researched the differences between CO-RADS scores from the publications available at the time of the study. Both evaluators were blinded to the clinical data and PCR results.

**Fig 1:**
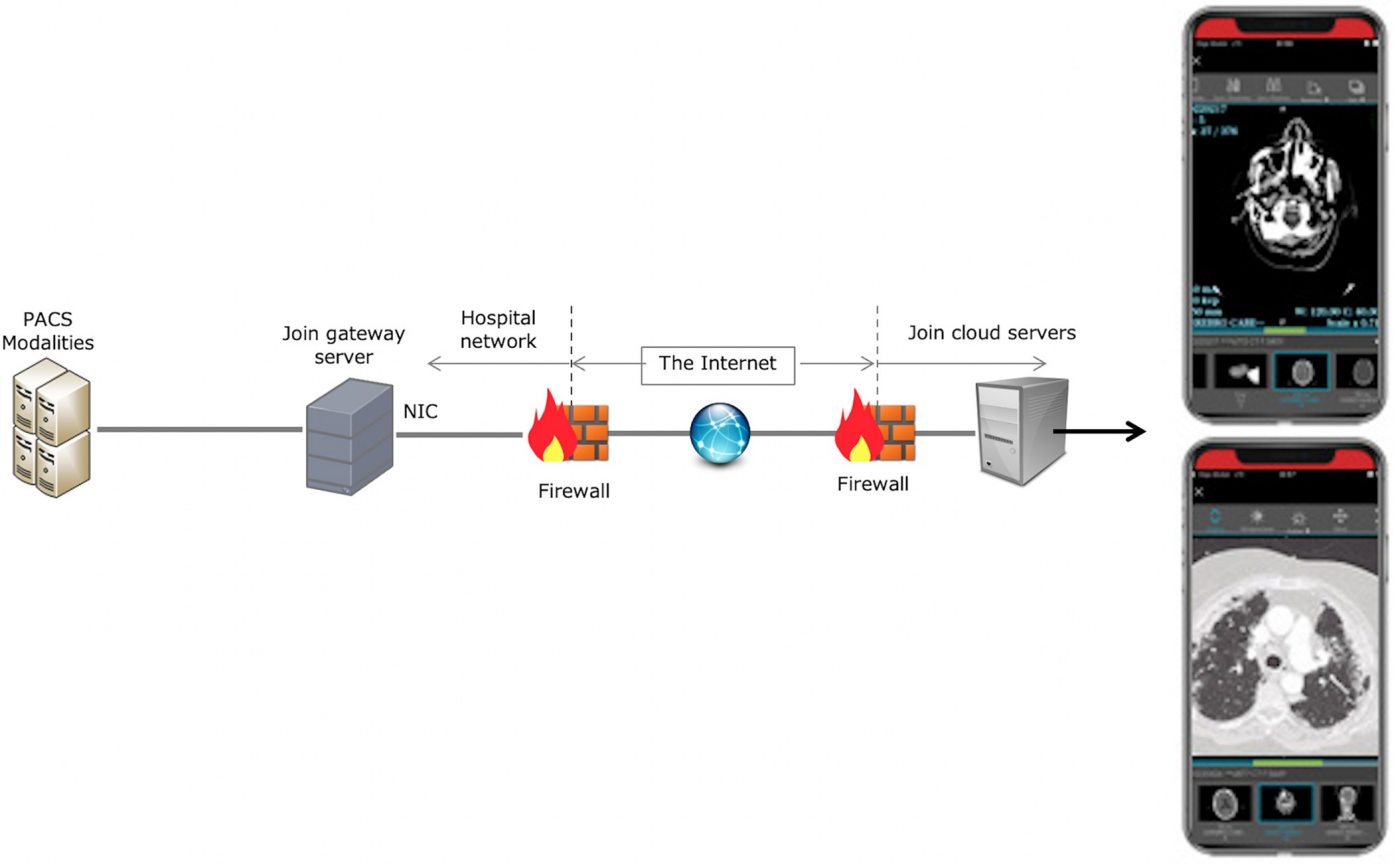
Example of encrypted image system for acute stroke code using the JOIN app.

For the second stage, we used the mobile app, JOIN (Allm, Tokyo, Japan), a certified communication app for health professionals designed to speed and optimize clinical communication and workflows, enabling the upload of radiology images to an encrypted server reachable by a standard smartphone connected to the Internet. The app also offers dedicated medical features such as an integrated DICOM viewer, timestamps, patient cases and chat and video telephony. JOIN is compliant with the European Union’s General Data Protection Regulation and ISO27001 and is certified by the US Food and Drug Administration (Fig 1).

A medical graduate analyzed the CTA source images using the JOIN app and PACS to evaluate COVID-19 apical lung findings, ranking the findings according to the CO-RADS scale. Image assessment using JOIN and the workstation was performed independently for each source, separated in time and in random order. These evaluators were also blinded to the clinical data and PCR results.

The study data were collected and managed using Research Electronic Data Capture (REDCap) [13] tools hosted at the IdiPAZ Health Research Institute. For the statistical analysis, we used IBM SPSS Statistics v21 (Chicago, IL, USA). Data are shown as absolute and relative frequencies for categorical variables or median and interquartile ranges for numeric variables. Data were compared using a chi-squared test, Fisher’s exact test, Student’s t test or Mann-Whitney’s U test, as appropriate. Concordance between the CO-RADS classification in CTA and chest CT, as well as the interrater agreement and concordance between the PACS and JOIN CO-RADS results were analyzed by Cohen’s kappa index (κ). The diagnostic performance of CTA for COVID-19 was based on the expert radiologist’s evaluation.

This study was approved by the Ethics Committee of La Paz University Hospital (PI-4660). As a retrospective study, the committee was exempted from requiring patient consent.

Patient and Public Involvement: The development of the research objectives and outcomes are based on the neurologist´s experience treating this profile of patients and the desire to optimise stroke care pathways in the COVID-19 pandemic waves. Patients and their advisors were not involved in the design, recruitment or conduct of this study.

## Results

**Fig 2:**
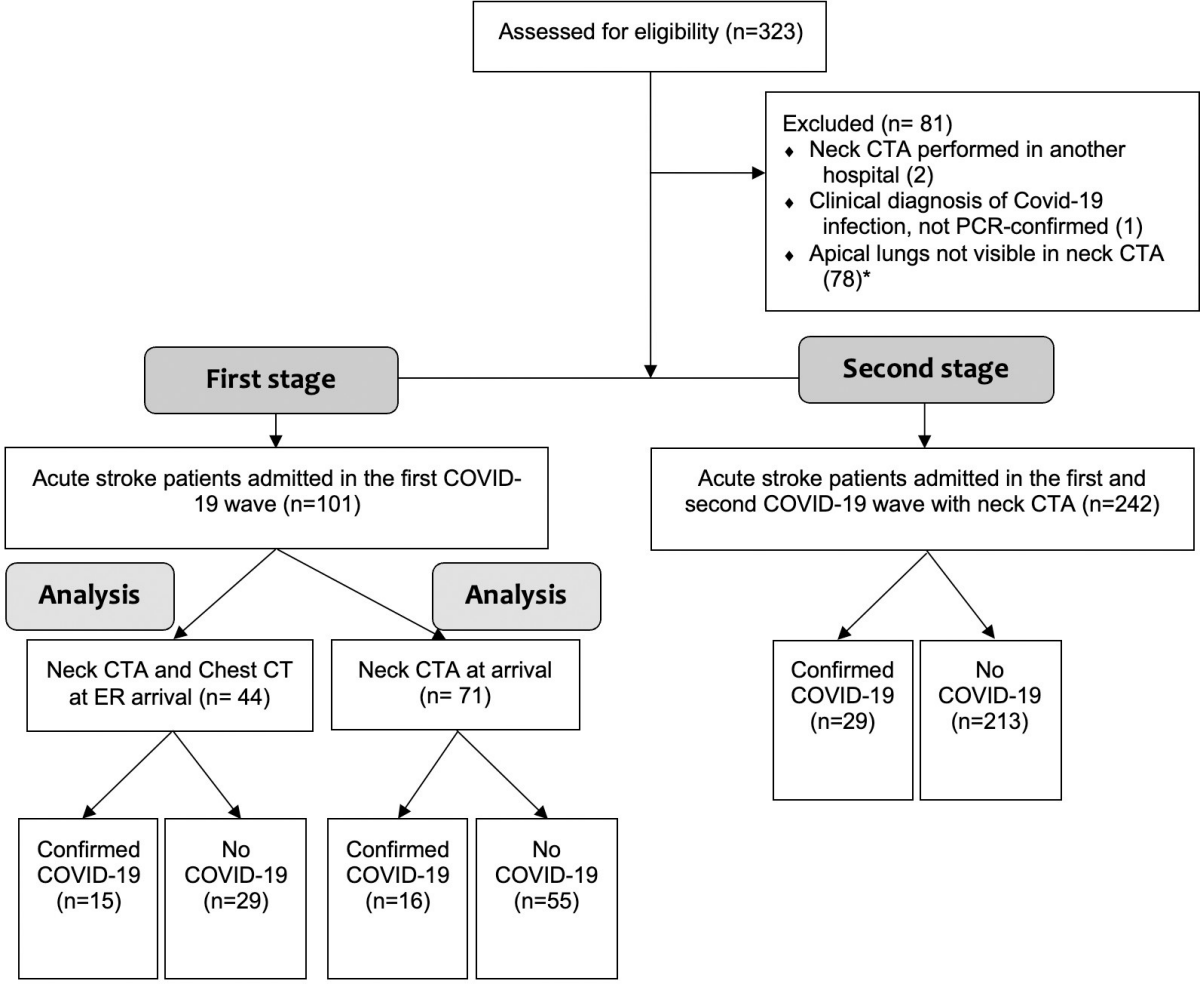
Study flowchart. Footnote: ER, emergency room; PCR, polymerase chain reaction. *excluded in the second stage.

A total of 323 patients with acute stroke were screened for inclusion in the various stages of the study, 81 of whom were excluded from the study due to various criteria. The sample selection is shown in the flowchart (Fig 2).

For the comparison between neck CTA and chest CT, 101 patients with acute stroke were included (24 with PCR-confirmed COVID-19). The cohort’s baseline characteristics are shown in Table 1.

**Table 1:** Demographics and baseline data in stage 1 and stage 2 cohorts.

	Stage 1				Stage 2			
	**Total sample**	**COVID-19**	**No COVID-19**	*P*	**Total sample**	**COVID-19**	**No COVID-19**	*P*
	N = 101	N = 24	N = 77		N = 242	N = 29	N = 213	
Age (years), mean (SD)	69.2 (14.5)	73.1 (10)	68 (15.4)	0.138	70.2 (15.1)	70.6 (12.2)	73.0 (15.4)	0.650
Male sex, n (%)	55 (54.5)	14 (58.3)	41 (53.2)	0.662	142 (58.7)	17 (58.6)	125 (58.7)	1
Hypertension, n (%)	66 (65.3)	18 (75)	48 (62.3)	0.255	153 (63.2)	20 (69.0)	133 (62.4)	0.545
Diabetes, n (%)	31 (30.7)	9 (37.5)	22 (28.6)	0.408	69 (28.8)	10 (35.7)	59 (27.8)	0.382
Atrial fibrillation, n (%)	18 (17.8)	6 (25)	12 (15.6)	0.293	48 (20.1)	8 (27.6)	40 (19.0)	0.322
COPD, n (%)	18 (17.8)	9 (37)	9 (11.7)	0.004	29 (12.1)	9 (20)	20 (9.5)	0.003
Tobacco use, n (%)	21 (20.8)	4 (16.7)	17 (22.1)	0.568	48 (20.2)	5 (17.2)	43 (20.6)	0.808
NIHSS at admission, median (IQR)	6 (3;15)	14 (5.2;20.5)	5 (2;13)	0.002	5 (2;13)	9 (5;20)	5 (2;11)	0.025
Chest CT performance, n (%)	53 (52.5)	18 (75)	35 (45.5)	0.011				
Neck CTA performance, n (%)	71 (70.3)	16 (66.7)	55 (71.1)	0.656				

COPD: Chronic obstructive pulmonary disease; IQR, interquartile range; NIHSS, NIH Stroke Scale

Both neck CTA and chest CT were performed on 43 patients, with 11 (25%) presenting lung findings suggestive of COVID-19. There was complete concordance in the classification according to CO-RADS between the two tests, with a COVID-19 diagnosis sensitivity and specificity of 53.8% and 92.9%, respectively, with a predictive positive value of 77.8% and negative predictive value of 81.3%.

**Fig 3:**
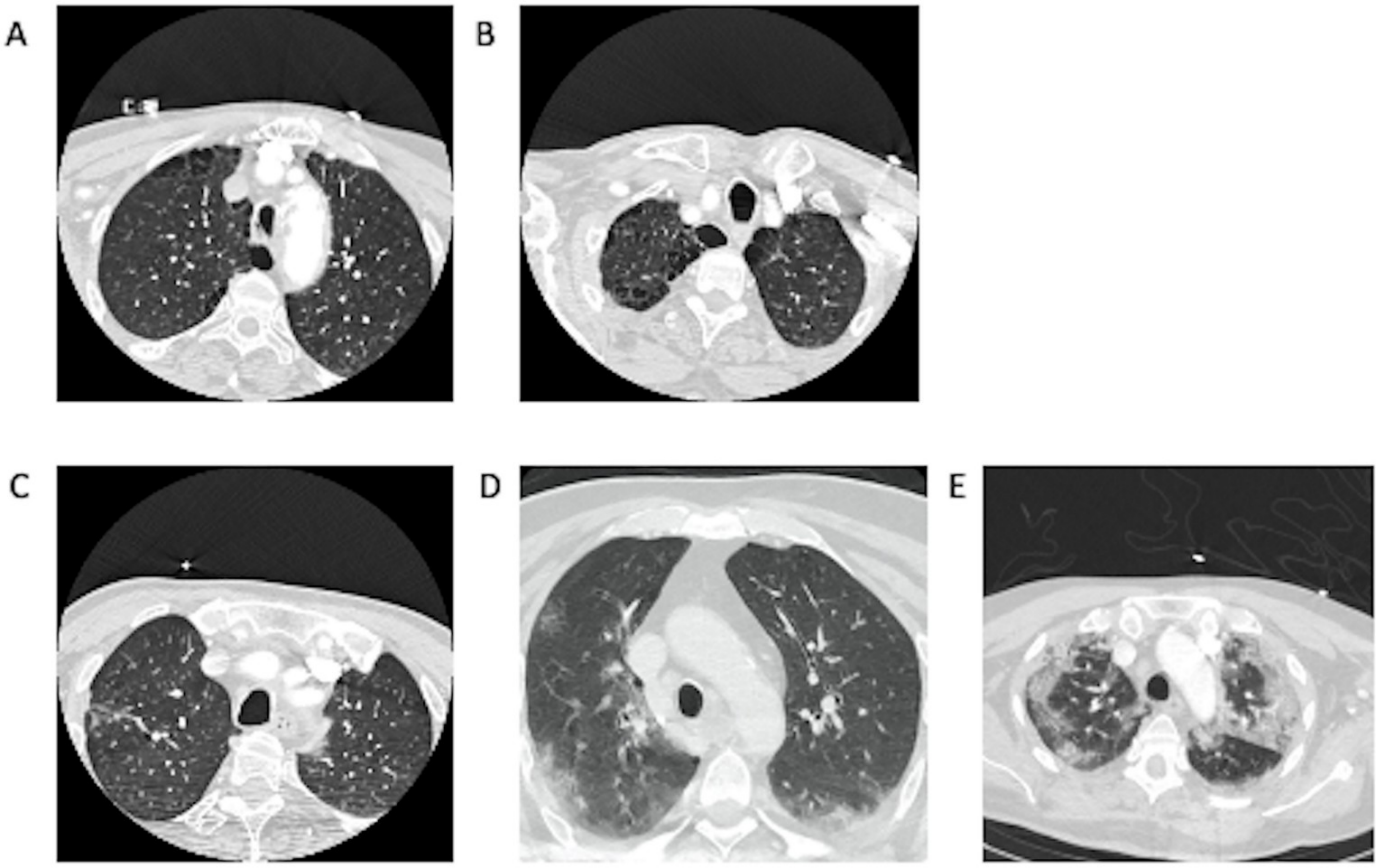
Examples for each COVID-19 Reporting and Data System (CO-RADS) score as evaluated in neck computed tomography angiography source image.

As a sensitivity analysis, we confirmed the predictive value in the cohort of 71 patients with neck CTA (sensitivity, 62.5%; specificity, 94.5%; positive predictive value, 76.9%; negative predictive value, 89.7%). Fig 3 shows examples for each CO-RADS score as evaluated by neck CTA.

The interobserver agreement in classifying the neck CTA lung findings into CO-RADS 3–5 or 1–2 was good (κ = 0.694; standard error, 0.107) (Table 2).

**Table 2:** Interrater agreement for the evaluation of COVID-19 lung involvement in neck computed tomography angiography.

	Rater 1	Rater 2
CO-RADS 1–2, n (%)	53 (76.8)	58 (81.7)
CO-RADS 3–5, n (%)	16 (23.2)	13 (18.3)

Footnote: K-Cohen = 0.694 SE 0.107

To compare the diagnosis performed in the workstations with that from the JOIN app, we included 242 patients with acute stroke who underwent neck CTA (29 with PCR-confirmed COVID-19). The cohort’s baseline characteristics are shown in Table 1. Confirmed COVID-19 patients had a higher frequency of COPD diagnosis and more severe strokes, with no significant differences in any other baseline characteristic.

The agreement in classifying the lung findings using the JOIN app and workstation with PACS into CO-RADS 3–5 or 1–2 was perfect (κ = 1.000; standard error 0.000) (Table 3).

**Table 3:** Intrarater agreement for the evaluation of COVID-19 lung involvement in workstations and JOIN.

	Workstation	JOIN
CO-RADS 1–2, n (%)	210 (86.8)	210 (86.8)
CO-RADS 3–5, n (%)	32 (13.2)	32 (13.2)

Footnote: K-Cohen = 1.000 SE 0.000

## Discussion

Several expert-based recommendations proposed performing chest CT combined with head-and-neck CTA in patients with acute stroke to rule out early COVID-19 and prevent contagion of attending personnel [3, 4, 14, 15]. Although the urgent acquisition of chest CT in acute stroke has suggested a benefit based on the quality-adjusted life years of patients and practitioners, its clinical impact might not be significant [16]. In contrast, routine chest CT screening for identifying COVID-19 pneumonia has been found to be of limited clinical value, particularly for asymptomatic patients or those who are screening within the first days of COVID-19 infection, and in intermediate-to-low COVID-19 incidence periods [17–19]. Therefore, chest CT is currently not recommended by most radiology societies [12] In addition, its performance might delay the workflow metrics in stroke management, especially at the start of mechanical thrombectomy in this sensitive time-dependent disease [6, 20].

To our knowledge, few studies have published the results of performing chest CT in patients with stroke during the pandemic. The correlation between acute neuroimaging abnormalities and a higher CT lung severity score has already been studied; however, the clinical implications of these findings have yet to be established [21]. Despite the high sensitivity of chest CT for diagnosing COVID-19 in patients with suspected stroke or head trauma (with a negative predictive value >93%) [22], a negative result does not rule out COVID-19 [12], which casts doubts on the cost-benefit value of including chest CT in stroke care protocols. Interestingly, neck CT that includes lung apices might show images consistent with COVID-19 pneumonia even in asymptomatic patients [23–25]. Indeed, Kihira et al. reported incidental COVID-19-related apical lung findings on CTA in up to 37.5% of patients with acute confirmed stroke, and the PCR for SARS-CoV-2 was positive in all of them [25].

Some authors have underscored the need to recognize COVID-19 features on radiographic images that may be acquired for other purposes to avoid overwhelming the healthcare systems in general, and radiology departments in particular [26]. To this end, our study showed that neck CTA performed as part of the routine assessment for patients with acute stroke is useful for identifying lung findings suggestive of COVID-19, with similar diagnostic performance as chest CT and good interrater agreement. Our findings therefore question the need for screening chest CT in patients with acute stroke to rule out COVID-19 lung involvement, which might help speed the workflow for treatment [3, 6]. Moreover, the good interrater agreement despite differences in terms of experience between the two readers supports the possibility that this new tool could be generally applied in emergency settings.

Although PCR tests are currently performed much faster, these diagnostic tests are not always available in certain settings, and, for a time-dependent disease such as stroke, any type of information that allows for the rapid detection of COVID-19 infection while waiting for PCR confirmation is of great utility in ensuring the proper protection of healthcare staff and other patients [6, 20, 24]. The importance of rapid information is emphasized during peaks of incidence.

This practice is especially important today, given that many hospitals are considering abandoning systematic screening tests for every patient who arrives at the emergency department, particularly if they show no symptoms of SARS-COV-2 infection. Considering that a number of COVID-19 patients are asymptomatic but can transmit the disease and that these patients would not be considered for PCR testing, our results highlight the value of apical lung evaluation in CTA source images in patients with stroke [12, 23]. However, this practice cannot replace the usual diagnostic tools, and in case of COVID-19 suspicion, a PCR test should be performed to confirm the infection.

Although a number of studies have shown the benefit of implementing mobile apps into the stroke code pathways [7, 8, 27, 28], this is the first to study its utility in detecting COVID-19 in patients with stroke. Our results show that the use of the JOIN app for this purpose is in complete agreement with the diagnosis performed with the PACS workstations, which suggests similar predictive values. Adding this information to the other relevant information for stroke code management using telemedicine in mobile apps might help speed all processes, especially when certain members of the stroke code are not always located in the hospitals and have to be activated by a stroke code call. This situation would be of particular relevance in collaborative systems for stroke care involving several hospitals in which certain patients might require interhospital transfer [7, 8, 27]. Any personnel involved in attending the patient even in different centers would have immediate information regarding the suspected COVID-19.

This study has certain limitations. First, this was a retrospective and unicentric study with a small sample size. Second, one of the raters was not an expert radiologist, which could have led to an underestimated interrater agreement. Third, to evaluate the concordance with Cohen’s kappa, the CO-RADS scale was reduced to a dichotomic option (possible or not possible COVID-19). Fourth, there is time-dependent variability in lung abnormalities caused by COVID-19, and the stroke date might not match the peak of lung involvement. Fifth, the involvement of upper lobes in COVID-19 is less frequent than in lower ones [26], and the frequency of chest CT findings in asymptomatic patients is less than 10% [18], which limits the negative predictive value of apical lung assessment in CTA source images; however, our study and others have demonstrated its positive predictive value in detecting COVID-19 apical lung involvement in approximately 25‒36% of acute stroke patients and in up to 59% of asymptomatic COVID-19 patients who underwent neck CT for different indications, including stroke [23, 25]. In addition, it has been recently reported that extending CTA to the full chest in acute stroke patients with unsuspicious upper chest scans only detects lower chest opacifications in about 13% of them [29]. Apical lung findings cannot replace confirmatory tests such as PCR but may help healthcare workers institute personal protective measures while waiting for the results of a confirmatory RT-PCR.

## Conclusions

Neck CTA is useful for the early detection of COVID-19-associated lung alterations in patients with acute stroke, with a predictive value comparable to that of chest CT and good interrater agreement. This evaluation can be performed through mobile apps such as JOIN, with almost complete agreement with the diagnosis reached in workstations. Although the absence of apical lung involvement in CTA cannot be used to rule out an asymptomatic SARS-CoV-2 infection, lung involvement findings using this readily available tool in stroke patients could be a red flag for healthcare workers to institute personal protective measures while waiting for the results of a confirmatory RT-PCR. Further studies are warranted to investigate the impact of implementing mobile apps to aid in the diagnosis of SARS-CoV-2 infection and timely communication to all stakeholders.
